# *Mayetiola destructor* (Diptera: Cecidomyiidae) host preference and survival on small grains with respect to leaf reflectance and phytohormone concentrations

**DOI:** 10.1038/s41598-021-84212-x

**Published:** 2021-02-26

**Authors:** Rohollah Sadeghi, Steven Odubiyi, Atoosa Nikoukar, Kurtis L. Schroeder, Arash Rashed

**Affiliations:** 1 Department of Entomology, Plant Pathology and Nematology, College of Agricultural and Life Sciences, University of Idaho, 875 Perimeter Dr., Moscow, ID 83843-2329 USA; 2 Department of Plant Science, University of Idaho, Moscow, ID USA

**Keywords:** Agroecology, Behavioural ecology

## Abstract

The Hessian fly *Mayetiola destructor* (Diptera: Cecidomyiidae) is a major pest of wheat, globally. We conducted a series of laboratory choice and no-choice assays to quantify Hessian fly host preference for barley (cv. Champion), oat (cv. Cayuse), susceptible (cv. Alturas), and resistant (cv. Hollis) wheat. In addition, larval survivorship and adult emergence were compared among the evaluated host plants. We then examined whether insect preference for a host can be explained by differences in plant spectral reflectance. Further, larval survivorship and adult emergence were compared among host plants in relation to phytohormone concentrations. Hessian flies laid more eggs on wheat compared to either oat or barley. Spectral reflectance measurements of leaves were similar between susceptible and resistant wheat cultivars but different from those of barley and oat. Our results suggested that higher reflectance in the near-infrared range and lower reflectance in the visible range may be used by females for host selection. Hessian fly larvae were unable to develop into the pupal stage on resistant wheat and oat. No significant difference in larval survivorship was detected between the susceptible wheat and barley. However, adult emergence was significantly higher on barley than the susceptible wheat. Phytohormonal evaluations revealed that salicylic acid (SA) may be an important contributor to plant defense response to larval feeding as relatively higher concentrations of SA were present in oat and resistant wheat. While resistance in the resistant wheat is achieved only through antibiosis, both antibiosis and antixenosis were in effect rendering oat as a non-host for Hessian flies.

## Introduction

Antibiosis and antixenosis are two mechanisms through which host plants defend themselves against arthropod pests. Antibiosis occurs when the host plant response negatively impacts one (or more) aspect the pest life history (e.g., egg reproduction, development and/or survival)^1–3^. Antixenosis is characterized as an adverse behavioral response from a would-be pest to some host plant stimuli^4^. Along with host tolerance, these resistance mechanisms have long been used to minimize damage by agricultural pests^5^.

The Hessian fly *Mayetiola destructor* (Say) (Diptera: Cecidomyiidae) is a critical global pest of wheat^6^, which has been effectively managed through planting resistant cultivars. In the Pacific Northwest (PNW) region of the USA, in the absence of host plant resistance, Hessian flies are expected to cause up to 10% yield loss in spring planted wheat^7^. In addition to wheat, many species of grasses (Poaceae) are also known to host Hessian flies^8^.

Adult female Hessian flies use physical^9–11^ and olfactory^11–14^ cues to locate their host for oviposition. Examples of physical stimuli include host plant leaf texture^10,12^, leaf age^9,12^ and spectral and spatial characteristics^11,15^. Some of these behavioral responses have been characterized in artificial laboratory setups to eliminate variability due to biotic and abiotic environmental factors^11,15^. For instance, Harris et al.^15^ demonstrated that Hessian flies positively responded to colored sheets with spectral reflectance ranging between 530 to 560 nm, but were repelled by the wavelengths in the 400 to 500 nm range. Although adult flies do not cause any direct feeding damage and live only for a few days, characterizing their behavioral responses to plant cues is critical as they perceive and utilize those stimuli to select hosts for oviposition. Hessian fly preference for wheat hosts has previously been demonstrated in New Zealand^8^.

Following egg hatch, the larvae move toward plant crown and, like other flies in the Cecidomyiidae family, form galls where they continue to feed and develop^16^. In wheat, feeding damage by the larvae can result in delayed plant development, dark green discoloration of the infested seedling, and subsequently, reduced yield and lodging due to weakened stem tissue^17^. After the completion of three larval instars^18^, a puparium is formed. Hessian fly puparium, also called “flaxseed”, is a developmental stage that enables the fly to survive harsh environmental condition (i.e., winter cold or summer heat). The duration of Hessian fly life cycle is primarily influenced by ambient temperature, with the possibility of having up to six generations per year in warmer regions^19–21^. In the Northern USA, with relatively shorter cropping seasons, no more than two generations are expected^22,23^.

Although, in the absence of control, larval feeding can hinder wheat productivity, there are currently at least 35 identified resistant genes (i.e., “R genes”), of which several have been incorporated into commercial cultivars^17,24,25^. Gene-for-gene interactions between the R gene in plant and an avirulence (*Avr*) gene or virulence (*v*) gene in herbivores define incompatible or compatible interactions, respectively. In incompatible interactions, plant defense responses are upregulated^26^. Plant defense involves three stages including surveillance (invasion detection), defense signaling, and the activation of specific defenses^27^. Through the surveillance stage herbivores’ attack signals are detected, and through a network of signaling pathways, defense strategies are activated^28^. Although the exact molecular mechanisms involved in the expression of an R gene have yet to be determined, it has been shown that the expression of an R gene can upregulate genes that encode insect toxins and are involved in cell wall metabolism^29^. These genes encode different proteins such as protease inhibitors, lectins, enzymes that produce phenylpropanoids, xyloglucan endotransglycosylases, β-expansins, pectinesterases, glucanases, cellulose synthases, dirigent-like proteins, class III peroxidase, lipid transfer proteins and a variety of lipase^30–34^. The activation of host plant defense prevents gall formation and promotes restoration of cell walls around feeding site, reducing nutrient availability to the larva^30^.

Phytohormones including jasmonic acid (JA), methyl jasmonate (MeJA), salicylic acid (SA), abscisic acid (ABA) and ethylene (ET) are signaling elicitors that regulate plant defense against biotic and abiotic stressors^35–39^. SA and JA/ET defense pathways have been reported to have antagonistic relationships^40,41^. JA regulates plant defense against herbivore with mechanical damage and necrotrophic plant pathogens^42^. SA on the other hand, regulates defense systems against pathogens and herbivores with piercing-sucking mouthparts, such as aphids^43^. Specifically, SA is known to upregulate gene expression related to lipoxygenases^26^, with potential role in plant defense against insects^44^. Hessian fly attack can lead to accumulation of SA, while small but significant reduction in JA was also observed for incompatible interaction between “R gene” and avirulent larvae^27^. This suggests that SA may play important roles in host plant resistance to Hessian flies. Similar to JA, ABA may also interfere with SA-dependent defense responses^45,46^ that contribute to Hessian fly resistance.

The present study was initially prompted by an anecdotal observation of a heavily damaged wheat variety trial planted within a healthy barley field in northern Idaho during a surge in Hessian fly population in the summer of 2019 (KS, personal observation). This observation suggested that the antixenosis in barley combined with resistant wheat as the trap crop could potentially and effectively divert damage away from barley and at the same time reduce Hessian fly pressure through antibiosis in resistant wheat. This of course would work, if similar to susceptible wheat, the resistant wheat is a preferred host for Hessian fly oviposition.

Using commonly planted PNW wheat (susceptible and resistant), barley and oat cultivars, and a laboratory colony established from local Hessian fly populations, we examined host preference (i.e., oviposition), larval survivorship and adult emergence rate on each of the host genotypes. Host preference and survivorship were quantified and discussed in relation to leaf spectral reflectance as well as phytohormone concentrations. Understanding of mechanisms that can predict the outcome of insect-host plant interactions is prerequisite to the development of sustainable and integrated pest management practices.

## Results

### Hessian fly host preference; choice assays

**Figure 1:**
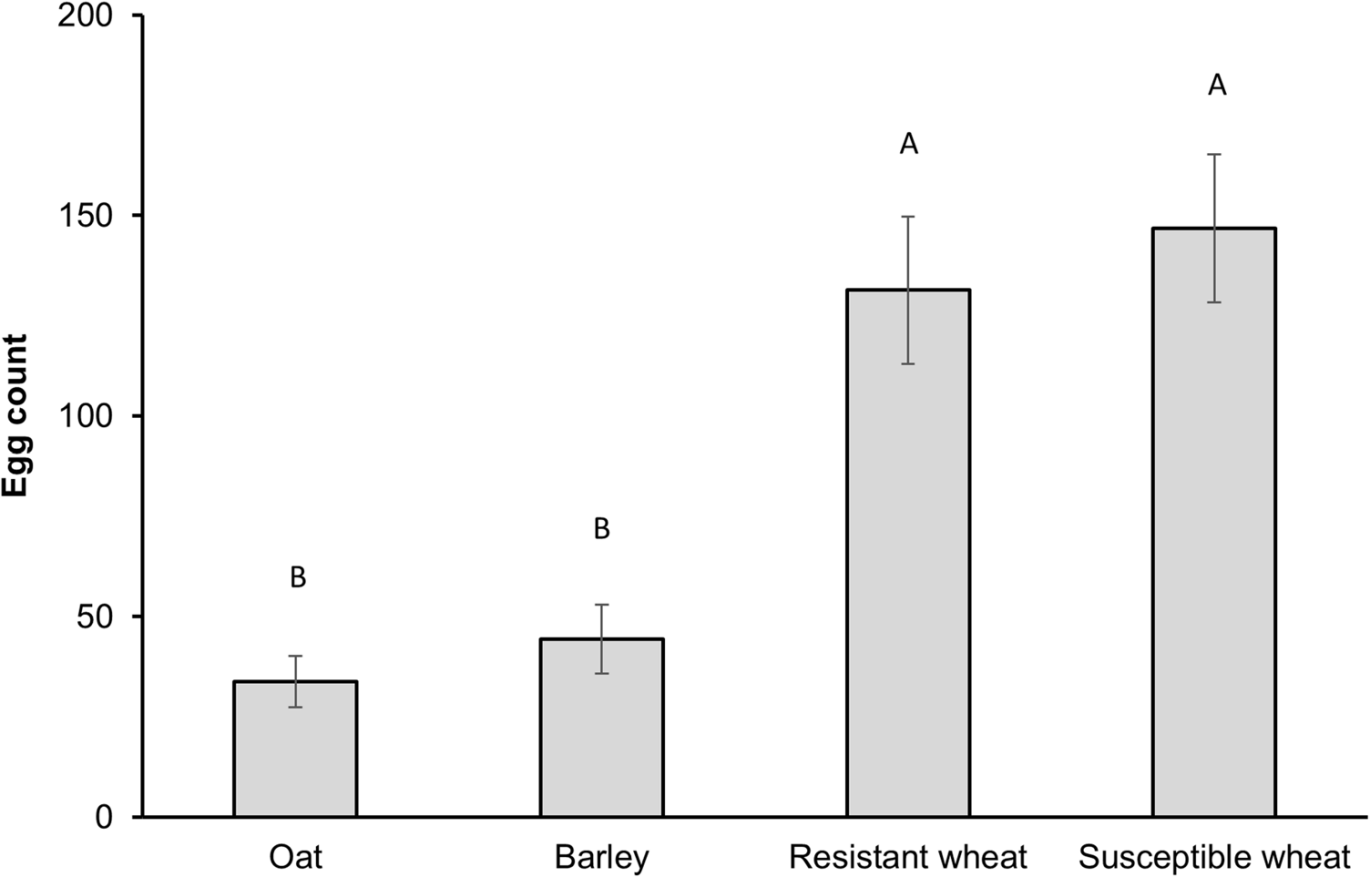
Oviposition (number of eggs per plant) by a single mated female on each host plant treatment in a choice test. Error bars represent standard errors (± SE). Oat (cv. Cayuse); Barley (cv. Champion); Resistant wheat (cv. Hollis); Susceptible wheat (cv. Alturas).

Hessian fly oviposition was significantly influenced by host treatment (GLMM, F_3,76_ = 17.02, *p* < 0.001). Although a significant effect of cage-replicate was present (F_*3,67*_ = 2.27, *p* = 0.028), no interaction was detected between host treatment and cage-replicate (*p* = 0.540). Fewer eggs were deposited on barley than either susceptible (*p* < 0.001) or resistant wheat (*p* < 0.001). Oat harbored the least number of eggs, which was significantly fewer than either wheat treatment (*p* < 0.001), but not barley (*p* = 0.332) (Fig. 1).

### Hessian fly host preference; no-choice assays

**Figure 2:**
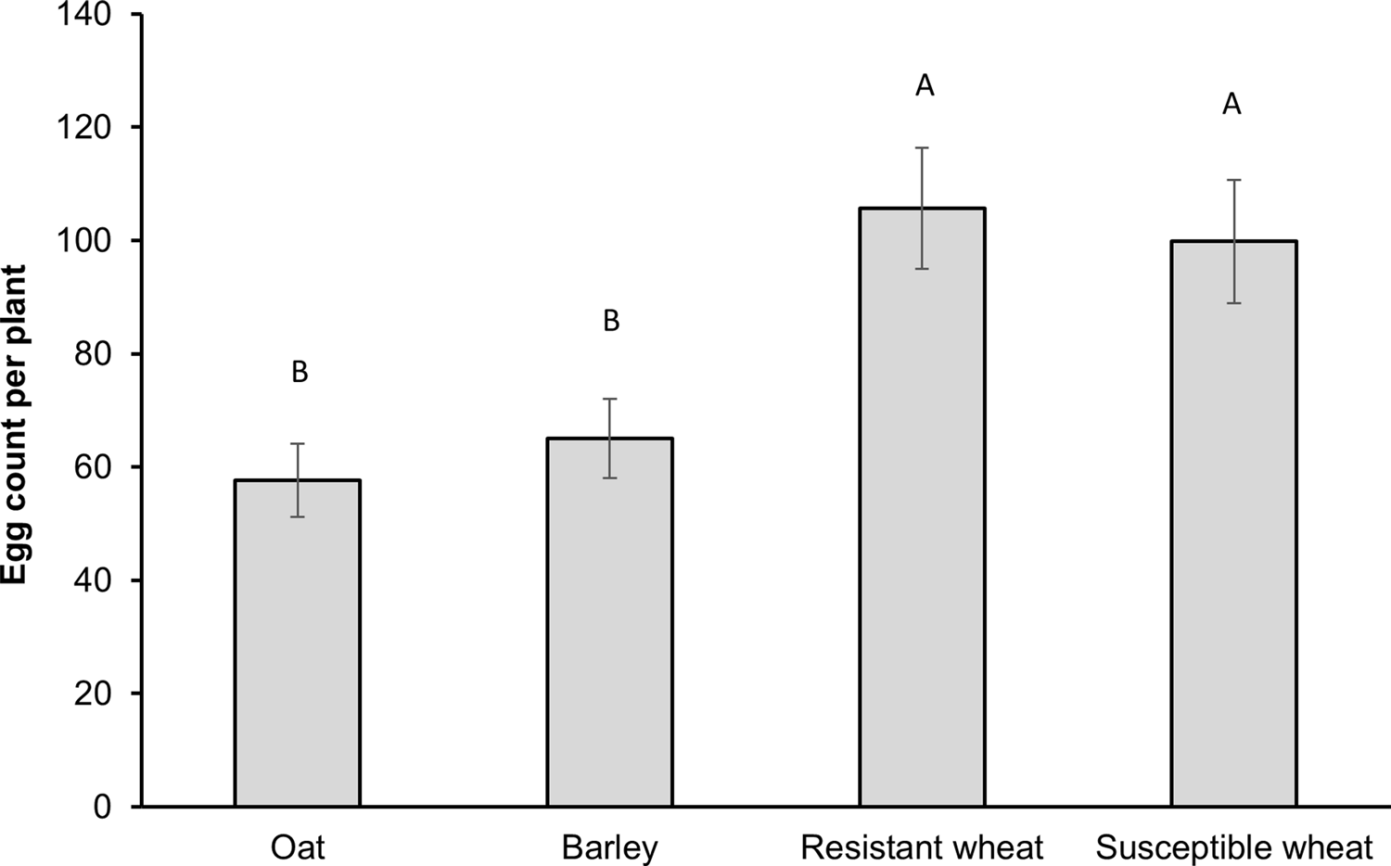
Oviposition (number of eggs per plant) by a single mated female on each host plant treatment in no-choice tests. Error bars represent standard errors (± SE). Oat (cv. Cayuse); Barley (cv. Champion); Resistant wheat (cv. Hollis); Susceptible wheat (cv. Alturas).

*The effect of host plant treatment on oviposition in no-choice assays:* Similar to our choice assays, in our no-choice assays the number of deposited eggs by the Hessian fly females was significantly affected by host treatment (GLMM, F_*3,108*_ = 6.80, *p* < 0.001; Fig. 2). Oviposition was significantly lower on oat when compared to both resistant (*p* = 0.001) and susceptible (*p* = 0.002) wheat cultivars. Likewise, barley harbored significantly fewer eggs than both the resistant (*p* = 0.005) and susceptible (*p* = 0.013) wheat cultivars. No statistical differences in oviposition was detected between the susceptible and resistant wheat cultivars or between oat and barley (Fig. 2).

**Figure 3:**
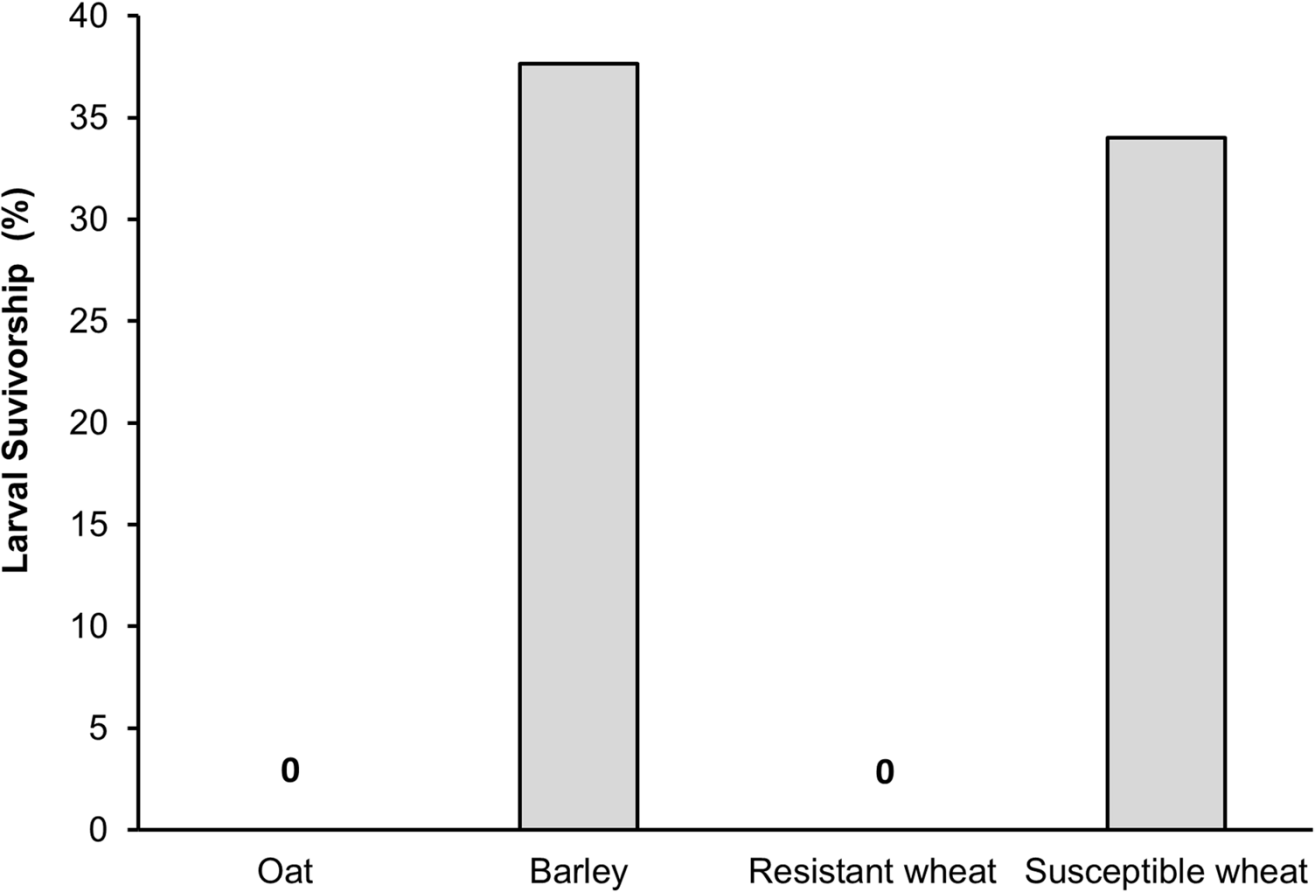
Survivorship of larvae on each host plant treatment in no-choice tests. None of the larvae made it to the pupal stage on either oat or resistant wheat host. Oat (cv. Cayuse); Barley (cv. Champion); Resistant wheat (cv. Hollis); Susceptible wheat (cv. Alturas).

**Figure 4:**
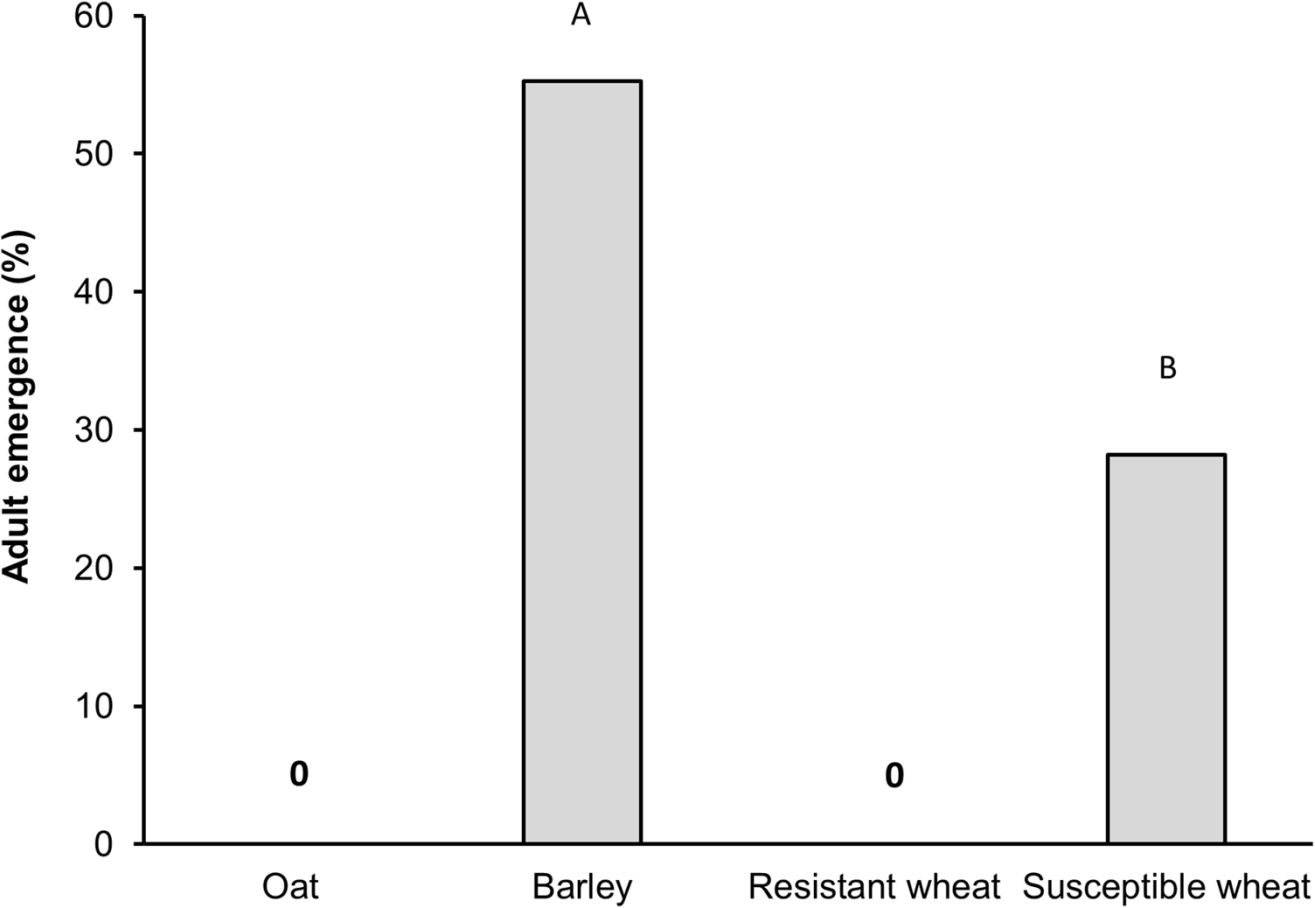
Percentage of adult emergence on each host plant treatment in no-choice test. Oat (cv. Cayuse); Barley (cv. Champion); Resistant wheat (cv. Hollis); Susceptible wheat (cv. Alturas).

*The effect of host plant treatment on larval survivorship and adult emergence in no-choice assays:* None of the Hessian fly larvae survived on either oat or the resistant wheat (Fig. 3). However, the larvae survived on both barley and the susceptible wheat and the larval survivorship was not statistically different between the two host treatments (F_*1,54*_ = 0.39, *p* = 0.536). Percent adult emergence, however, was significantly higher on barley (55%) compared to the susceptible wheat, Alturas (28%) (F_*1,22*_ = 6.7, *p* = 0.017) (Fig. 4).

### Leaf adaxial surface reflectance analysis

**Figure 5:**
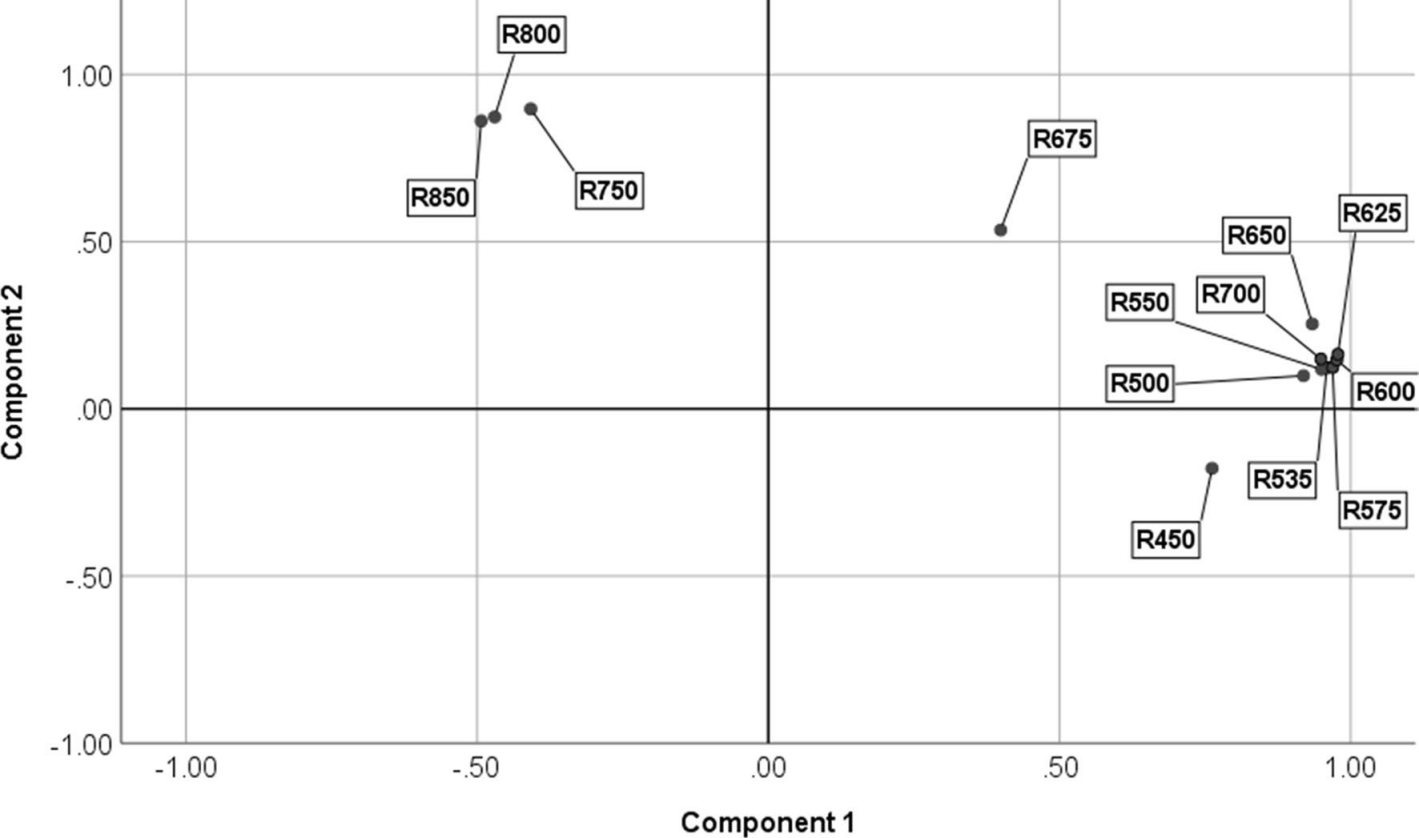
Variable correlation plot for the first two principal components extracted by PCA. Variables are reflectance at the selected thirteen wavelengths. R450 through R850 represent the reflectance measurements at 450 through 850 nm wavelengths.

**Figure 6:**
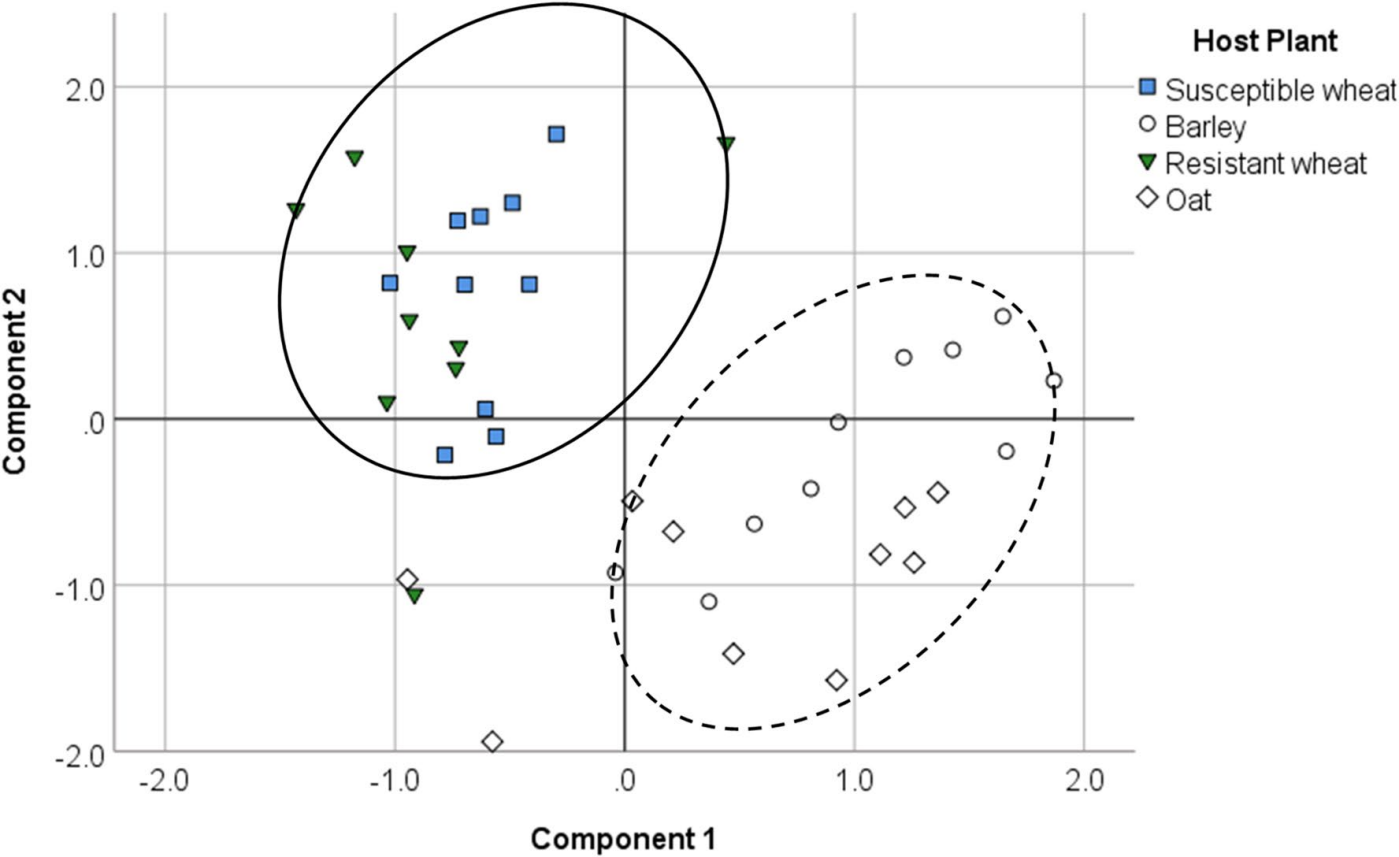
PCA plot of the first two components of the individual spectrum based on the host plant treatments. Solid line indicates the relative position of wheat cultivars and dashed line indicates relative position of barley and oat within plot area. Oat (cv. Cayuse); Barley (cv. Champion); Resistant wheat (cv. Hollis); Susceptible wheat (cv. Alturas).

The first and second PCA components explained 67% and 22% of variation in reflectance measurements, respectively. Component 1 showed strong positive correlations with reflectance at 500, 535, 550, 575, 600, 625, 650, 700 (Corr. Coefficient *r* > 0.92), and 450 nm (*r* = 0.76). Component 1 (PC1) also showed weak negative correlations with reflectance at 750 nm (*r* = -0.41), 800 nm (*r* = -0.47) and 850 nm (*r* = -0.49). Component 2 (PC2) was strongly positively correlated with reflectance at 750, 800, and 850 nm (*r* > 0.86) (Fig. 5). Both susceptible and resistant wheat cultivars were positively associated with the reflectance in the near-infrared range (750–850 nm), which were correlated with PC2, and negatively associated with the reflectance in the visible range (400–700 nm), which showed correlation with PC 1 (Fig. 6). Both susceptible and resistant wheat cultivars were categorized in the same group, distinct from the barley and oat cultivars. Barley was grouped with oat showing positive association with PC1, and negative association with PC2 (Fig. 6). Barley placement was positively associated with PC1, and positively or negatively associated with PC2.

### Phytohormones in response to Hessian fly infestation

The three evaluated phytohormones ABA, SA and JA were detected and quantified in both infested and non-infested plants. Phytohormone concentrations in the stem tissue were influenced by both host treatment (MANOVA; Pillai’s Trace: F_*9,192*_ = 9.49, *p* < 0.001) and infestation status (Pillai’s Trace: F_*3,62*_ = 9.49, *p* < 0.001). In barley, ABA and JA concentrations increased significantly in the infested compared to the non-infested plants, effects which were not detected in the other evaluated host plant treatments (Table 1). In oat, SA concentration was significantly reduced in the presence of the larvae, however it was still numerically higher than the SA concentrations recorded in other host treatments (Table 1). In the resistant wheat, the increase in SA in the presence of the larvae was of borderline significance (*p* = 0.062). Due to a significant interaction between infestation status and host plant treatment (Pillai’s Trace: F_*3,192*_ = 9.71, *p* < 0.001), we proceeded to analyze non-infested and infested treatments, separately.

**Table 1:** Effect of infestation on the concentration of phytohormones in the host plant treatments.

Host Plant	PH	Phytohormone concentration (ng/g FW) ± SD		*P* value
		Non-infested	Infested	
Oat (cv. Cayuse)	ABA	7.80 ± 1.10	8.26 ± 0.99	n.s
	SA	38.98 ± 18.54	18.34 ± 6.35	< 0.001
	JA	9.12 ± 0.96	9.88 ± 1.72	n.s
Barley (cv. Champion)	ABA	7.19 ± 0.54	9.00 ± 2.60	0.029
	SA	5.49 ± 1.40	6.64 ± 1.92	n.s
	JA	7.93 ± 0.66	11.87 ± 3.27	< 0.001
Susceptible wheat (cv. Alturas)	ABA	8.67 ± 0.77	8.48 ± 0.68	n.s
	SA	7.43 ± 2.15	11.35 ± 3.23	n.s
	JA	9.63 ± 0.73	9.47 ± 1.04	n.s
Resistant wheat (cv. Hollis)	ABA	7.39 ± 0.94	7.56 ± 0.69	n.s
	SA	6.09 ± 1.10	16.79 ± 7.81	0.062*
	JA	8.06 ± 1.06	9.12 ± 1.18	n.s

PH: Phytohormone, ABA: abscisic acid, SA: salicylic acid, JA: jasmonic acid.

n.s.: Nonsignificant.

*Nonsignificant but biologically relevant for comparisons.

**Figure 7:**
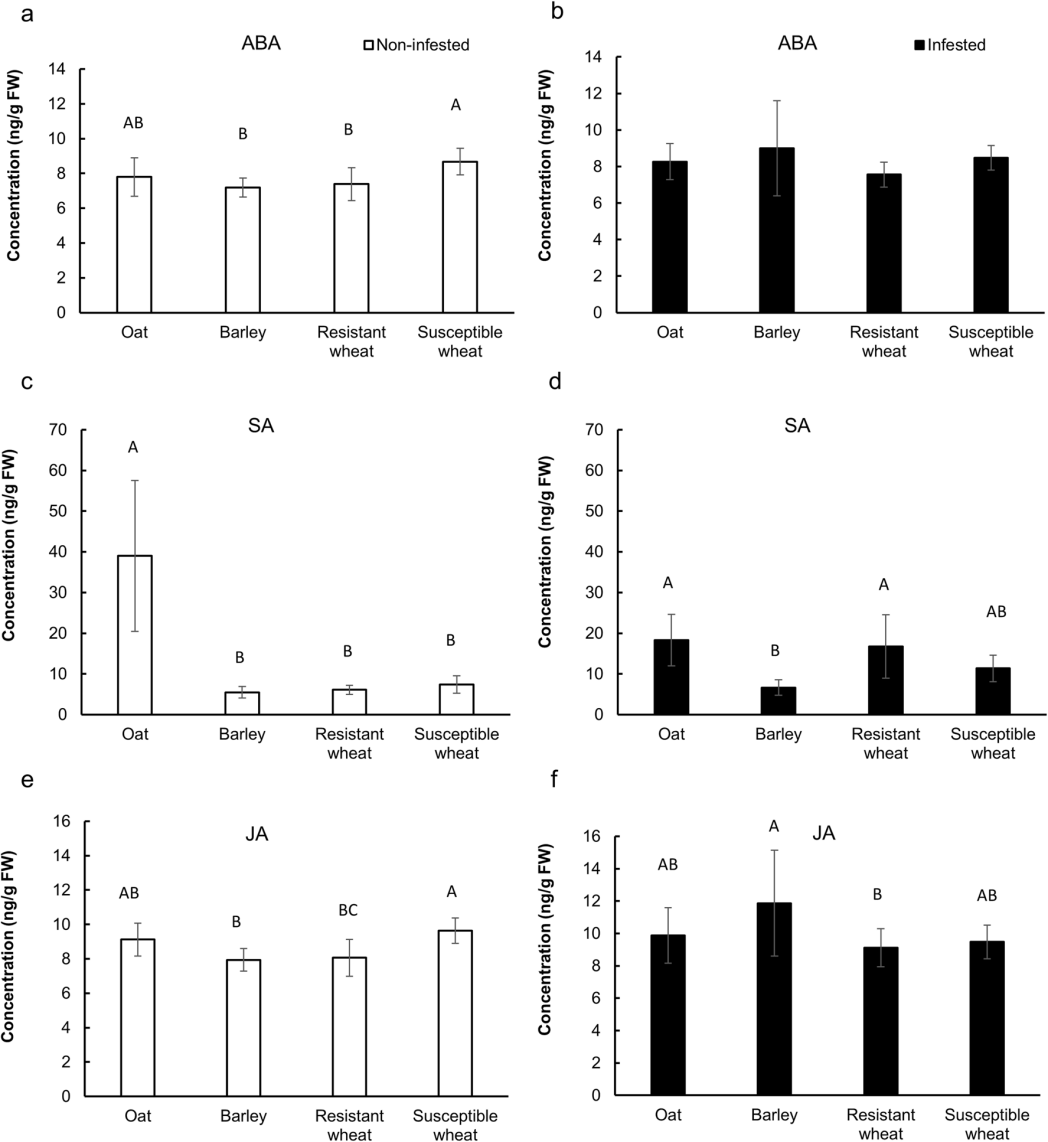
Concentration of abscisic acid (ABA) (**a** and** b**), salicylic acid (SA) (**c** and** d**), and jasmonic acid (JA) (**e** and** f**) in the host plant treatments in non-infested (left: white column) and infested (right: black column) hosts (Oat (cv. Cayuse); Barley (cv. Champion); Susceptible wheat (cv. Alturas); Resistant wheat (cv. Hollis)). Error bars represent standard deviation (± SD).

In non-infested host plants, phytohormone concentrations varied significantly among host treatments (Pillai’s Trace: F_*9,102*_ = 7.82, *p* < 0.001). ABA concentrations were significantly different between the resistant (Average concentration ± SE: 7.39 ± 0.30 ng/g FW) and susceptible (8.67 ± 0.24 ng/g FW) wheat cultivars (*p* = 0.01) as well as between barley (7.19 ± 0.17 ng/g FW) and the susceptible wheat (*p* = 0.002). However, oat and barley (*p* = 0.42), barley and resistant wheat (*p* = 0.95), and oat and either wheat cultivars (*p* > 0.13) formed homogenous subsets according to Tukey HSD pairwise comparisons (Fig. 7). SA concentration in oat (38.98 ± 6.55 ng/g FW)) was significantly higher than barley (5.49 ± 0.44 ng/g FW) and the two wheat cultivars (resistant: 6.09 ± 0.35 ng/g FW; susceptible: 7.43 ± 0.68 ng/g FW) (*p* < 0.001). Barley, the resistant and the susceptible wheat formed a statistically homogeneous subset (*p* = 0.160) (Fig. 7). JA concentration was only significantly different between barley (7.93 ± 0.21 ng/g FW) and susceptible wheat (9.63 ± 0.23 ng/g FW).

In infested host plants, phytohormone concentrations were significantly different across host treatments (Pillai’s Trace: F_*9,99*_ = 4.39, *p* < 0.001). This overall variation was primarily due to variations in JA (F_*3,33*_ = 3.23, *p* = 0.035) and SA (F_*3,33*_ = 6.37, *p* = 0.002) concentrations. The JA concentration was only significantly different between barley (11.87 ± 1.09 ng/g FW) and resistant wheat (9.12 ± 0.39 ng/g FW) (*p* = 0.024); no significant differences were observed between other host treatments. Higher concentrations of SA were present in oat (18.34 ± 1.41 ng/g FW) and the resistant wheat (16.79 ± 1.74 ng/g FW). Lower concentrations of SA were detected in barley (6.64 ± 0.38 ng/g FW) and the susceptible wheat (11.35 ± 0.81 ng/g FW). ABA concentrations were not affected by host treatment (F_*3,33*_ = 1.40, *p* = 0.259).

## Discussion

Through a series of laboratory choice and no-choice assays, we demonstrated that the preference of the PNW Hessian flies for selected PNW oat, barley and wheat genotypes is nonrandom, and that the observed resistance to the Hessian flies in some of the evaluated host plants can be achieved through antibiosis, antixenosis, or a combination of both mechanisms.

Our choice assays indicated that barley (cv. Champion) and oat (cv. Cayuse) are less attractive to the Hessian fly females compared to either resistant (cv. Hollis) or susceptible (cv. Alturas) wheat. Antixenosis was clearly in effect as Hessian flies deposited approximately 3 to 4 times more eggs on wheat than either oat (cv. Cayuse) or barley (cv. Champion). The presence of antixenosis was further confirmed when flies were not given the choice to select their host plant for oviposition. Higher egg numbers were recorded for both the resistant and susceptible wheat in no-choice assays, up to 1.8 times more than those recorded on oat and barley. Despite using different host cultivars, our results supported those by Harris and colleagues^47^ in that wheat is generally a more preferred host of Hessian flies than oat and barley.

The preference of the gravid Hessian flies toward wheat as their oviposition host can be explained, at least in part, by variations in visual cues. Reflectance measurements from both wheat cultivars in the visual range were negatively correlated with PC1 (Fig. 6). This, and the observed positive correlation between wheat near-infrared reflectance measurements and PC2 are reflective of plant health and a vigorous seedling development. Our findings supported Harris et al.^15^ who reported that the green range wavelengths (530 nm to 560 nm) are generally attractive to the Hessian flies. It has been demonstrated that the total leaf chlorophyll content is associated with the increased ratio of reflectance at 750 nm wavelength (near infrared) to that at 550 nm wavelength (green)^48^. The reduced reflectance in this visual range is indicative of sufficient light absorption by leaf pigments, which in combination with high reflectance at near-infrared wavelengths, would signal a healthy host suitable for supporting the development of the Hessian fly offspring. The relatively lower reflectance at the near-infrared range and higher reflectance at the visual range observed in barley and oat may be perceived by the gravid Hessian flies as a sign of unpalatability, resulting in reduced oviposition. It is also important to note that insects are known to use a combination of visual and olfactory cues to select their host plant^10,12–14,49^. In relation to this, it has been demonstrated in an artificial set up (i.e., colored sheets and plants extracts) that Hessian flies use a combination of tactile, color and olfactory cues to select their host; removing either tactile or color cue reduced oviposition by 70%, whereas removing chemical cues reduced the number of deposited eggs by 93%^11^. In the present study, however, we did not assess differences in olfactory cues among host plant treatments. Future studies with actual host plants are needed to further demonstrate the importance of both visual and olfactory cues on Hessian fly host preference.

Although in our choice assay, barley was one of the least preferred hosts, it supported larval development just as well as the susceptible wheat, a preferred host of the Hessian flies. Interestingly, adult emergence was significantly higher on barley than on the susceptible wheat. In conjunction with results from choice assay, it can be concluded that the resistance in barley is only achieved through antixenosis. The anecdotal field observations of Hessian fly damaged wheat within intact barley fields combined with our results, suggest that in high risk regions planting barley fields with wheat as the trap crop may help to reduce damage. Although, to date, Hessian flies have not been a serious pest of barley, there are reports of barley impacted by this pest in the Pacific Northwest (Dr. M. Pumphrey, personal communications) and other USA regions (e.g., Georgia)^50^.

No flaxseeds were present on either oat or resistant wheat, indicating a 100% larval mortality on both host treatments. These results support those of Harris et al.^47^ in New Zealand. This between-study consistency indicates that oat is nonhost for the Hessian fly. There is, however, a closely related species, *Mayetiola avenae* (Marchal) reported to infest oat in Tunisia^51^. As for wheat, however, breakdown of resistance and/or partial resistance may occur; continuous planting of a resistant cultivars with a single ‘R gene’ is expected to facilitate this breakdown^52–54^. Rotating resistance varieties with different R genes is expected to relax selection pressure on Hessian fly biotype elongating the effectiveness of the R gene^52,55^.

While larval feeding is prerequisite to its survival and later pupal formation, feeding on plants expressing defensive physiological responses are known to result in larval toxicity^29^. Phytohormones are known signaling elicitors regulating plant defensive responses^56^. In the present study, the level of phytohormones in both non-infested and infested plant treatments was evaluated to understand the roles of the phytohormones in host plant defense against Hessian fly larvae. In non-infested plants, both JA and ABA concentrations were higher in the susceptible wheat compared to resistant wheat. Moreover, significant increases in the concentrations of ABA and JA were observed in infested barley. However, higher concentrations of ABA and/or JA did not appear to contribute to the reduced larval survivorship and adult emergence, in particular, since these phytohormones were not elevated in the resistant hosts, oat and resistant wheat (cv. Hollis). In non-infested plant treatments, SA concentration was significantly higher in oat than other plant treatments. Although SA concentration in oat was significantly reduced when infested, it remained relatively higher than the other evaluated host plants. Indeed, the increase in SA concentration in the infested relative to the non-infested resistant wheat was of borderline statistical significance (*p* = 0.06), but noteworthy. Our finding suggested that SA is likely the phytohormone that is associated with oat and resistant wheat defense against Hessian flies. Our results supported those of Zhu et al.^27^, who suggested a key role for SA in wheat resistance to Hessian flies. It is possible that higher concentrations of SA in oat and the resistant wheat mediate the release of defense related compounds, detrimental to Hessian fly development, such as proteinase inhibitors^32^, carbohydrate-binding proteins^33,57^ and phenylpropanoids like *trans*-cinnamate ^30^ as well as reactive oxygen species (ROS)^58^. However, unlike Zhu et al.^27^, no antagonistic relationship between SA and JA was detected in our study. JA is known to upregulate defense against chewing insects^42^ and the increase in SA concentrations in the infested hosts plants, such as resistant wheat, may be explained by the Hessian fly larvae feeding mechanism. The larva inserts its mandibles into the epidermal cells and feeds on plant sap through the damaged tissue after injecting saliva^59^; this feeding mechanism is more similar to feeding by the piercing-sucking insects than insects with chewing mouthparts.

In summary, our findings from choice and no-choice assays indicated that resistance to Hessian fly presence in barley is only through antixenosis, which would be most effective when a preferred host, such as wheat, is also present. One might argue that choice findings do not necessarily indicate antixenosis but rather a preference toward wheat. The observed reduced oviposition on barley in no-choice assays however supported the existence of antixenosis. Moreover, as resistance in wheat is solely conferred through antibiosis, using resistant wheat as trap crop is expected to contribute to reduced pest populations. Oat was the only evaluated host that was resistant through both antibiosis and antixenosis. The present study was focused on only one commonly planted cultivar of oat, barley, resistant and susceptible wheat in the PNW region. Complementary studies, using the most cultivated regional small grain varieties, and local populations of Hessian flies, can confirm and generalize these findings. A clear understanding of traits that can contribute to tolerance and resistance and their underlying mechanisms of expression is essential for facilitating the development of new resistant cultivars and integrated pest management strategies in small grain production systems.

## Materials and methods

### Insect colony and plants

Hessian flies used in all experiments were from laboratory colonies established from field-collected puparia during the summer of 1998 in Lewiston, ID, USA^60^. Colonies are reared on the susceptible spring wheat cultivar “Alturas”, at 21 ± 4 °C, with a 16:8 L(light):D(dark) photoperiod. Hessian fly population structure in Lewiston, around the time of laboratory colony establishment, was made of multiple biotypes, with the majority belonging to the two biotypes, F (38%) and GP (25%)^61^.

Only gravid females were used in host preference and oviposition assays. Gravid females can be distinguished by larger body size (due to swollen abdomen), reduced mobility, as well as the absence of mate calling behavior (rhythmical exposure of the ovipositor/genitalia) typical of the unmated Hessian flies.

Two wheat (*Triticum aestivum*) cultivars, the susceptible “Alturas” and the resistant “Hollis”, the barley (*Hordeum vulgare)* cv. Champion and the oat (*Avena sativa)* cv. Cayuse were used in this study. Hereafter, these plant types are referred to as “host treatment”. The wheat seed were provided by the University of Idaho Wheat Breeding Program at the Aberdeen Research and Extension Center, Aberdeen, ID, and Washington State University Wheat Breeding Program, Pullman, WA. Barley and oat seed were provided by the University of Idaho, Cropping Systems Agronomy Program in Moscow, ID. All seed were planted individually in 10 × 10 cm plastic pots filled with a blend of 85.5% standard germination growth mix (Sunshine mix #1, Sungro Horticulture Canada Ltd, Seba Beach, Canada), 14.2% sand, and 0.3% Osmocote smart-release plant food (14:14:14), moistened with water.

### Hessian fly host preference; choice assays

Plants were at the two- or three-leaf stage at the time of the experiment. Each assay consisted of four plants, one from each host treatment, randomly arranged inside a 50 × 50 × 50 cm transparent plexiglass cage with two sides covered with see-through mesh. There was a total of 10 cage-replicates in each of the two time-blocks.

Four gravid females from the laboratory colony were released into each cage (one female/seedling). The four host treatments were exposed to the female flies for a 24-h period, after which plants were removed and the number of eggs deposited on adaxial and abaxial surfaces of the leaves were counted under a stereomicroscope (Wild-Heerbrugg, Heerbrugg, Switzerland). The number of eggs on each host treatment was used to determine Hessian fly host preference.

### Hessian fly host preference; no-choice assays

The no-choice assays were setup to compare oviposition, larval survival and egg-to-adult emergence on each of the four host treatments, barley, oat, and susceptible and resistant wheat. Plants were seeded as previously described in choice assays. In no-choice assays, however, each potted plant was contained individually using cylindrical plexiglass cages (22 × 5 cm, H × D). Each plant was infested with one gravid female at the 2-leaf stage.

The caged plants were arranged in trays in a complete randomized design. There was a total of 6 time-blocks, with either four (time-blocks 1 through 4) or six (time-blocks 5 and 6) plant-replicates per host treatment. All six time-blocks were used for comparing oviposition and larval survival among host treatments (112 experimental units). The last two time-blocks were continued until adult emergence, with a total of 48 experimental units (see ‘adult emergence success’).

*The effect of host plant treatment on oviposition and larval survivorship in no-choice assays:* After the 24-h exposure time, the cylindrical cages were removed and the number of eggs on each plant was counted under a stereomicroscope. Plants were then randomly arranged in the large 50 × 50 × 50 cm plexiglass cages. The access panels (i.e., mesh sides) in the front and back of each cage were covered with plastic wraps for 5 days in order to retain humidity (RH 50–85%) for egg incubation, after which plastic covers were removed.

Twenty-one days after infestations, plants were removed and dissected to report the number of flaxseeds. Larval survivorship on each seedling was estimated by dividing the number of flaxseeds by the number of eggs deposited on each seedling. Flaxseed weight was not significantly different on the susceptible wheat and barley cultivars (data not shown).

*The effect of host plant treatment on adult emergence in no-choice assays:* Twenty-one days post infestation, individual plants were removed from large cages, placed in plastic trays and then covered with cylindrical cages. Adult emergence was monitored for a period of 14 days, starting 28 days after plant infestations. The number of puparia cases was counted and emergence success was recorded based on the percent emergence, calculated by dividing the number of adults by the number of flaxseeds, multiplied by 100.

### Host plant reflectance analysis

The reflectance measurements at visible (VIS) and near infrared (NIR) ranges were conducted on the adaxial surface immediately after removing the leaf. Ten individual plants were selected at the 2-leaf stage (14 days after planting). For each host plant, the middle portion of the youngest leaf was measured at three points each 5 mm apart to collect representative leaf spectra. A Jaz-S spectrometer (Ocean Optics, Winter Park, Florida, U.S.A.), equipped with one 400-mm VIS–NIR reflection probe (covering 340–1032 nm range), was used for the spectra collection and analysis. The probe was stabilized at a 45° angle, 2 cm above the leaf surface using a probe holder. All reflectance measurements were conducted in a dark room, and the light source was covered with a black cloth to minimize external light interference, the spectrometer was calibrated against a diffuse reflectance standard (Labsphere’s Spectralon®, Labsphere, North Sutton, NH) and background noise was collected and corrected by fully blocking the light source output. Spectra were initially visualized, and data was exported to spreadsheets using SpectraSuite Spectroscopy Software (Ocean Optics, Largo, FL). An average spectrum of each host plant treatment was plotted and visually compared to find the most critical wavelengths for further analysis (Electronic Supplementary Material-Figure S1). Thirteen wavelengths including 450, 500, 535, 550, 575, 600, 625, 650, 675, 700, 750, 800, and 850 nm were selected for principal component analysis (PCA; see ‘statistical analysis’). These wavelengths are also known to be reflective of leaf pigmentation (e.g., chlorophylls and carotenoids) and other chemical attributes like nitrogen content^62–64^ as well as leaf structure including leaf thickness, geometry and orientation^64^.

### Host plant phytohormone analyses

Salicylic acid (SA), jasmonic acid (JA) and abscisic acid (ABA) in each of the host plants, were quantified in both non-infested plants and plants infested with the Hessian fly larvae. To initiate the experiment, twenty plants per host treatment were seeded individually in pots, as previously described. At the 2- to 3- leaf stage, ten plants per host treatment were infested by releasing 10 gravid females into 50 × 50 × 50 cm plexiglass cages, each containing 10 potted seedlings (one female/seedling). Each host treatment was infested in a separate cage. The other 10 plants per host treatment were placed in separate 50 × 50 × 50 cm plexiglass cages, where they remained non-infested. The oviposition and egg hatching were monitored. Twenty-four hours after hatching of all the eggs (~ 10 days after infestation), plant stems were collected individually, weighed, placed in 2 ml screw cap cryotubes with three 2.8 mm ceramic beads (Omni International Inc., Kennesaw, GA), and frozen in liquid nitrogen. The non-infested host treatments were also sampled at the same time.

All samples were ground using an Omni Bead Ruptor 12 homogenizer (Omni International Inc., Kennesaw, GA) for 60 s at high speed. Then, 1 mL of the extraction solvent (methanol:water; 7:3) with 150 ng internal standard (dihydrojasmone) was added to each tube for extraction according to Almeida et al.^65^. The samples were ground again for 30 s at low speed, and extraction was done for 30 min using a Vortex Genie 2 (FisherScientific, Bohemia, NY) at speed 8. The tubes were centrifuged at 21000 g for 10 min at 4 °C, and the supernatant was removed and transferred into a 1.5 mL microtube. The pellets were washed with 200 μL of methanol and the supernatant was added to the corresponding microtubes. The extracts were dried in a SpeedVac Concentrator (Savant Instruments Inc. Farmingdale, NY), and re-dissolved in 100 μL methanol for the analysis. An average of the two individual samples were used for statistical analysis as independent replicates.

The phytohormone analyses were performed using a high-performance liquid chromatography (HPLC) system (Agilent 1200 Series) with a diode array detection (DAD) system coupled to an Agilent G1969A TOF–MS system equipped with an ESI source (Agilent, Santa Clara, CA, USA). A Zorbax XDB-C18, 50 mm × 4.6 mm, 1.8 µm column (Agilent, Santa Clara, CA, USA) was used for the separation holding at 30° C. The analysis was done according to Davis et al.^66^ method using 0.1% formic acid in water as solvent A and 0.1% formic acid in methanol as solvent B. The gradient program started with a linear gradient from 50 to 95% B in 5 min, then followed by isocratic elution at 95% B for 2 min, and finally equilibrated at 50% B for 3 min. Quantification was performed in the reconstructed ion current mode using 263.13 m/z for ABA, 137.02 m/z for SA, and 209.11 m/z for JA.

### Statistical analysis

Host choice assays were analyzed using a generalized linear mixed model (GLMM) with host treatment, cage replicate and the interaction between the two as main effects and time-block as random effect. The interaction term was excluded from the final model due to its non-significant effect. A negative binomial distribution with an identity link function was applied.

Egg number, larval survivorship, and adult emergence in no-choice assays were analyzed using a GLMM with host treatment as the fixed and time-block as the random factor. Normal distribution was assumed for larval survival and adult emergence percentages. A negative binomial distribution, with an identity link function was applied to egg counts. Since none of the larvae survived on oat, or the resistant wheat, they were not included in the statistical analyses for larval survivorship or adult emergence.

Principal component analysis (PCA) was used to interpret reflectance data for the thirteen selected wavelengths and to group host treatments in a two-dimensional plot based on two principal components.

Multivariate analysis of variance (MANOVA) was conducted to determine whether concentrations of ABA, JA, and SA are influenced by the host infestation status (infested vs non-infested), host treatment and the interaction between host treatment and infestation status. Due to the presence of a significant interaction, infested and non-infested host plants were analyzed separately. MANOVAs were followed up by univariate analysis of variance (ANOVA) to decouple the effect of each factor on individual phytohormones. Post hoc Tukey HSD tests were used to conduct pairwise comparisons between host plants.

All data were analyzed with IBM SPSS Statistics ver. 26.0.

## Supplementary Information

Supplementary Information 1.

## Data Availability

The data used to support the findings of this study are available from the corresponding author upon request.
