# Author Correction: *Mayetiola destructor* (Diptera: Cecidomyiidae) host preference and survival on small grains with respect to leaf reflectance and phytohormone concentrations

**DOI:** 10.1038/s41598-021-90074-0

**Published:** 2021-06-07

**Authors:** Rohollah Sadeghi, Steven Odubiyi, Atoosa Nikoukar, Kurtis L. Schroeder, Arash Rashed

**Affiliations:** 1 Department of Entomology, Plant Pathology and Nematology, College of Agricultural and Life Sciences, University of Idaho, 875 Perimeter Dr., Moscow, ID 83843-2329 USA; 2 Department of Plant Science, University of Idaho, Moscow, ID USA

Correction to: *Scientific Reports* https://doi.org/10.1038/s41598-021-84212-x, published online 26 February 2021

The original version of this Article contained errors where “Cecidomyiidae” was incorrectly given as “Cecidmyiidae”. This resulted in an error in the title.

Additionally, in the Abstract,

"The Hessian fly Mayetiola destructor (Diptera: Cecidmyiidae) is a major pest of wheat, globally."

now reads:

"The Hessian fly Mayetiola destructor (Diptera: Cecidomyiidae) is a major pest of wheat, globally."

These errors have now been corrected in the PDF and HTML versions of the Article, and in the accompanying Supporting Information file.

