# Supplementary material for: Mayetiola destructor (Diptera: Cecidomyiidae) host preference and survival on small grains with respect to leaf reflectance and phytohormone concentrations
Source: Sci Rep. 2021 Feb 26;11:4761. doi: 10.1038/s41598-021-84212-x (PMC7910616; doi:10.1038/s41598-021-84212-x)
Supplement: Supplementary file 1 — Supplementary Information 1. [file 41598_2021_84212_MOESM1_ESM.docx]

**Hessian fly (*Mayetiola destructor,* Diptera: Cecidmyiidae) host preference and survival on small grain crops with respect to leaf reflectance and phytohormone concentrations**

**Rohollah Sadeghi^1^, Steven Odubiyi^1^, Atoosa Nikoukar^1^, Kurtis Schroeder^2^, Arash Rashed^1*^**

^1^Department of Entomology, Plant Pathology and Nematology, University of Idaho, Moscow, ID

^2^Department of Plant Science, University of Idaho, Moscow, ID

*Running title: Host plant resistance to Hessian fly*

Corresponding author: Arash Rashed, University of Idaho, College of Agricultural and Life Sciences, Department of Entomology, Plant Pathology and Nematology, 875 Perimeter Dr., Moscow, ID 83843-2329 USA; Email: [arashed@uidaho.edu](mailto:arashed@uidaho.edu); Tel: 208-885-5972

Journal of Pest Science

Electronic Supplementary Material

Figure S1: Average reflectance spectra of the leaf surface of oat (cv. Cayuse), barley (cv. Champion), susceptible wheat (cv. Alturas), and resistant wheat (cv. Hollis) host plants. Each spectrum is an average of three scans of ten leaves in two-leaf stage seedlings.
